# Exploring the experiences and needs in everyday life of spouse carers of persons with dementia

**DOI:** 10.1080/17482631.2026.2680726

**Published:** 2026-05-27

**Authors:** Marcus F. Johansson, Lena Marmstål Hammar, Lena Dahlberg, Kevin McKee, Chirstine Williams, Martina Summer Meranius

**Affiliations:** a School of Health and Welfare, Dalarna University, Falun, Sweden; b Department of Health Science, Innovation and Design, Faculty of Engineering and Health Sciences, Mälardalen University, Västerås, Sweden; c Aging Research Center, Karolinska Institutet & Stockholm University, Solna, Sweden; d Christine E Lynn College of Nursing, Florida Atlantic University, Boca Raton, FL, USA

**Keywords:** Informal care, dementia, spouse, support need, couple relationships

## Abstract

**Purpose:**

Spouses of persons with dementia often take on caring responsibilities that can be overwhelming and negatively affect their well-being. To support the development of effective services and promote carer well-being, we aim to describe carer experiences and needs in everyday life.

**Methods:**

Semi-structured phone interviews were conducted with a convenience sample of 24 spouses caring for a partner with dementia in Sweden. The interviews explored experiences of caring for a partner with dementia. Interviews were analyzed with thematic analysis.

**Results:**

The analysis generated two themes: “Being consumed by caring” and “Longing to be seen and feel supported”. The first theme showed that spouse carers experience a transition from spouse to carer and feel confined in their new situation. The second showed that to be supported, spouse carers need to feel acknowledged in their situation, and formal care must meet their partner's needs, as carers' needs are enmeshed with those of their partners.

**Conclusion:**

Spouse carers of persons with dementia often feel trapped by caring responsibilities. To feel supported, they need to be acknowledged both as individuals and as part of a couple. Health and social care professionals should focus on maintaining spouses' sense of self and adopt a couple-centred approach.

## Introduction

Dementia is a leading cause of disability and dependency. It most commonly occurs in people over the age of 65, affects over 57 million people worldwide, and often results in a need for care and support in the person with dementia's everyday life (GBD Dementia Forecasting Collaborators, 2022; World Health Organization, 2017). Health and social care sectors across the globe face difficulties in meeting the long-term care needs of an ageing population (Barreira et al., 2023). In Sweden, a majority of persons with dementia reside in the community with a spouse as a primary source of care and support (Socialstyrelsen, 2022; Wimo et al., 2020). Caring for a person with dementia is typically stressful and can lead to negative outcomes such as depression, which are more prevalent in spouse carers than other sub-groups of informal carers (Johansson et al., 2021; Kirvalidze et al., 2023; Zhu et al., 2024).

The caregiving experience of a spouse carer of a person with dementia has some notable aspects that are due to the nature of their relationship with the care-recipient. Spouse carers commonly live with their partner with dementia (Johansson et al., 2021), and many will have been in a relationship with that partner for most of their adult life (Johansson et al., 2022; Pope et al., 2025). They often find it hard to accept their partner's dementia, seeking alternative explanations for their partner's changing behaviour. When their partner's dementia can no longer be denied, they may experience grief, loneliness, isolation and fear from the thought of losing their life companion (Egilstrod et al., 2019; Kotwal et al., 2024; Pope et al., 2025; Zhu et al., 2024). Spouse carers may feel worried and uncertain regarding the future, both for themselves as an individual and also in relation to their partner and their carer role (Greenwood et al., 2019; Kotwal et al., 2024; Zhu et al., 2024) and can experience their caring responsibilities as overwhelming (Macdonald et al., 2020). However, spouse carers may also experience caring as positive, finding it rewarding, meaningful, and enhancing personal and emotional growth, as well as bringing them closer to their partner (Johansson et al., 2022; Quinn et al., 2024; Zhu et al., 2024).

Persons with dementia tend to have progressive and changing needs over the course of the disease (Black et al., 2019). Consequently, the care provided by spouses and how it is experienced will evolve with the nature and severity of their partner's dementia (Miller et al., 2025; Pope et al., 2025; Zhu et al., 2024). Spouse carers of persons with dementia often fulfil their caring responsibilities alone (Johansson et al., 2021; Ornstein et al., 2019), and may have a broad range of support needs (Johansson et al., 2024; Mansfield et al., 2023; Miller et al., 2025).

Spouse carers may also perceive that, with increasing care responsibilities, their spousal relationship changes (Miller et al., 2025; Steenfeldt et al., 2021; Zhu et al., 2024). This transition, where a person acquires an identity as a carer, has been described by Montgomery and Kosloski (2009, 2013) within caregiver identity theory. The acquisition of a carer identity has generally been seen as inevitable for spouse carers (Morgan et al., 2021), with the result that they may find it hard to navigate between their own needs and their partners care needs (Clemmensen et al., 2020; Morrisby et al., 2019; Steenfeldt et al., 2021). Consequently, spouse carers may neglect their own interests and health and perceive themselves as hostages to perceived expectations to care (Macdonald et al., 2020; Miller et al., 2025; Shiff et al., 2025).

As such, support for spouse carers of persons with dementia should ideally address their perceived needs in their specific social situation, yet studies indicate that spouse carers often report being offered support that they neither desire nor need (Hammar et al., 2019; Tyrrell et al., 2019; Zhang et al., 2025). While several systematic reviews indicate that commonly offered forms of support (e.g. counselling, support groups, carer proficiency training) have poor or uncertain effects on reducing the negative impact of caring (Abrahams et al., 2018; Balvert et al., 2024; Kirvalidze et al., 2023), some suggest they may provide value for spouse carers with regard to obtaining relief, experiencing affinity with other carers and fostering a sense of reciprocity between carer and care-recipient (Kirvalidze et al., 2023; Williams et al., 2019). Research has found that spouse carers of persons with dementia, as well as their partners prefer support to be flexible and tailored to meet their unique and changing needs as individuals as well as couples, and more research is required to understand how the support needs of carers could be met (Bannon et al., 2022; Pope et al., 2025; Van Aerschot et al., 2022).

While several studies have investigated the life situation and burdens of being a spouse carer of a person with dementia, more research is needed on their experiences: not only of their perceptions of need for support and of the support received in caring for a partner, but also of their everyday life; as both an individual and when considered as one member of a couple. The findings of such research would add to the evidence needed to develop and deliver appropriate and effective support. The aim of this study is to describe the life- and caring situation of spouse carers of persons with dementia and explore their experiences and needs of support in everyday life. The aim has been operationalized in the following research questions:

- How do spouse carers experience their everyday life in relation to their caregiving?
- When do spouse carers feel supported and what are their further needs of support in caregiving?

## Methods

### Design

This study employed a qualitative design with individual semi-structured interviews and thematic analysis.

### Sampling and participants

Participants were selected from spouse carers who participated in a previous study on spousal care of people with dementia in Sweden (Johansson et al., 2022). The original study consisted of a convenience sample of 163 carers, self-identified based on the following definition: “this study concerns you, a person aged 65 years or older and living with a spouse or partner with a dementia disorder to whom you provide care, help or support”. In addition, the eligibility criteria in the original study included proficiency in Swedish.

In this study, all individuals (61 females, 15 males) who agreed to be contacted for follow-up studies were sent an invitation to take part in an interview. Potential participants were informed that the interview would concern their experiences of caring for, and living with, a partner with dementia, how they were supported, and their need for further support. In total, 24 persons responded to the invitation and were interviewed.

### Data collection

A semi-structured interview guide was developed by the research team based on the findings of a pilot study (Hammar et al., 2019) and previous research. The full interview guide is provided in Supplementary Material S1. The interview guide was divided into different areas: relationship, life situation, future and support. Each area contained a variation of open-ended questions. Interviews were conducted by phone due to COVID-19 recommendations on social distancing. The interviews lasted from 25 to 131 min and were audio-recorded and transcribed verbatim.

### Data analysis

The interview transcripts were analyzed using thematic analysis following Braun and Clarke's approach (Braun & Clarke, 2022), which offers structure to the process of identifying, analyzing, and reporting patterns of meaning in qualitative data. In the first stage of the analysis, transcripts of the interviews were read thoroughly to gain familiarity with the data by three of the authors (MFJ, MSM, LMH). During this process, impressions and initial observations, potential patterns and interpretations of meaning related to the study aim were noted and discussed. The interpretations were based on both a semantic understanding, that is, the explicit meaning as expressed in the content of the data, and a latent understanding, that is, the underlying concepts and assumptions influencing the overt content of the data.

In the following stage of the analysis the first author re-read all transcripts, providing each of the observations in the transcripts with labels describing their meaning (codes). During this process, codes for observations with a similar meaning where merged or modified, resulting in a reduced number of analytic codes based on both semantic and latent meanings. After this, the codes were organized based on patterns of meaning into themes relevant to the study's aim.

The process of generating themes from transcripts was iterative, meaning that transcripts were repeatedly re-read with observations re-coded and subthemes and themes organized, until co-authors agreed on the meaning and pattern of the observations. The iterative process provided a deeper understanding of the themes and the overall structure which resulted in collapsing seven initially identified candidate themes into two themes with subthemes. During this process potential names for themes and subthemes were discussed in relation to the study aim.

### Ethical statement

The study was conducted in accordance with the Declaration of Helsinki and approved by the Swedish Ethical Review Authority (reg. no. 2019-03288, 2020-02987) in adherence with the Swedish Ethics Review Act (2006: 460). All participants were given oral and written information concerning the study. They were also informed that participation was voluntary; that informed consent was required and that they had the right to withdraw from the study without offering an explanation; that results would be pseudonymized and no statement would be linked to specific individuals; that topics in the interview could be considered private or of a sensitive nature, and they could chose to be interviewed by a female or male member of the research team; that the interview was audio recorded; that by consenting to, and participating in the study consented for results to be published in scientific journals. In addition, they were informed on the rules for processing personal data contained in the EU General Data Protection Regulation (2016/679) and the Swedish Public Access to Information and Secrecy Act (2009:400); and that they had the right to get their data corrected or deleted. Prior to participation, written informed consent was obtained from each participant. When quotes are presented in the results, all participant's names have been assigned pseudonyms.

## Results

Anonymized participant characteristics are summarized in Table I. At the time of recruitment, the carers were aged between 65 and 89 years, with a mean age of 73.6; the mean age of the care receiving partners was 74.8. Most carers were women who cared for a man (*n* = 17), with seven male carers, of whom six cared for a woman. All couples resided in ordinary housing and were retired. Half of the care-receiving partners had a diagnosis of Alzheimer's disease (*n* = 12).

**Table I.** Participant characteristics.

Participant	Gender	Age	Gender partner	Age partner	Duration of relationship	Dementia diagnosis	Years as carer
Sarah	Female	65	Male	70	46	Alzheimer's disease	5
Dorothy	Female	65	Male	66	46	Unspecified	10
Patricia	Female	65	Male	67	43	Dementia with Lewy Bodies	4
Helen	Female	66	Male	73	19	Alzheimer's disease	1
Barbara	Female	69	Male	76	51	Alzheimer's disease	-
Gerda	Female	70	Male	72	46	Alzheimer's disease	3
Irene	Female	71	Male	73	50	Dementia with Lewy Bodies	3
Monica	Female	72	Male	71	49	Alzheimer's disease	5
Sylvia	Female	73	Male	74	57	Alzheimer's disease	3
Karen	Female	73	Male	72	28	Alzheimer's disease	1
Arthur	Male	74	Female	72	52	Alzheimer's disease	7
Jane	Female	74	Male	73	31	Vascular dementia	6
John	Male	74	Female	72	65	Alzheimer's disease	6
Hilda	Female	74	Male	74	8	Vascular dementia	4
Catherine	Female	75	Male	75	50	Under evaluation	3
Edna	Female	75	Male	73	40	Unspecified	4
Martha	Female	75	Male	81	51	Frontotemporal dementia	3
Maureen	Female	75	Male	80	42	Alzheimer's disease	2
Brigitt	Female	75	Male	78	53	Vascular dementia	5
Stephen	Male	77	Male	67	35	Alzheimer's disease	5
Sam	Male	77	Female	76	57	Alzheimer's disease	5
George	Male	78	Female	81	60	Unspecified	12
Gerald	Male	86	Female	88	67	Vascular dementia	10
Clifford	Male	89	Female	92	63	Vascular dementia	7

The thematic analysis generated two themes to capture participants' descriptions of their life situation and their needs in everyday life (Table II). The first theme, *Being consumed by caring* contains two subthemes: *Navigating the new role and loss of companionship* and, *Feeling confined and forsaking own needs.* The second theme, *Longing to be seen and feel supported* also contains two subthemes: *Valuing acknowledgement in one's individual situation* and *Needing support suitable for us.*

**Table II.** Main themes, sub-themes, and example observations from transcripts.

Theme	Subtheme	Example extract
Being consumed by caring	Navigating the new role and loss of companionship	“I think about things like tools, taking care of the house, and such, that's always been my husband's domain. I feel quite... well, all thumbs, so to say. You know, because he's the one who has taken care of [the house etc.] ... and when I try to get him to help… I get so frustrated when he doesn't understand what I mean.” (Dorothy, 65)
	Feeling confined and forsaking own needs	“I'm becoming sentimental now with an upcoming wedding; we can't travel. We travelled in 2018; that was the last time. But now it's starting to be like that, it's the kind of thing I give up. So, that's what it is... it's something I can't do; it's not my own time; all time is dedicated to my husband, he comes first, so to speak.” (Sarah, 65)
Longing to be seen and feel supported	Valuing acknowledgement in one's individual situation	“There is someone who calls me [from the social services] every other week, they ask how I am doing and if there is anything else they can do for me. That is very good, and she [the social worker] has visited our house and checked up on me as well.” (Clifford, 89)
	Needing support suitable for us	“I don’t know if I could enjoy myself and relax if I knew he was in a place where he doesn't know anyone, and they don't know him and such. It wouldn't have felt right...” (Patricia 65)

### Being consumed by caring

As their partner's dementia progressed, participants described how they were increasingly consumed by their caring role and had less time to spend with friends or on personal interests. They became less of a spouse and more of a carer, with everyday life conditioned by their partner's dementia.

#### Navigating the new role and loss of companionship

Participants had to find new ways to navigate everyday life with uncertainties related to their partner's dementia diagnosis. Commonly, they tried to live in the moment to enjoy the time they had remaining with their partner; unwilling to contemplate their own or their partner's future. John said in response to a question on how he perceived his future:

“Well, I… I push it ahead of me. You’ll cross that bridge when you come to it… It is nothing that bothers me. Nothing is like the present, and I will enjoy and live in the present.” (John, 74)

As their partner's dementia progressed, participants tried to maintain their spousal role and relationship while often experiencing a loss of both companionship and intimacy. The perception of transitioning roles in the couple was often driven by changes in the division of household responsibilities as they compensated for their partner's loss of function. This sometimes required that the participant acquire new skills needed to manage the household alone.

While managing new household responsibilities brought a sense of accomplishment, navigating their changing role in their relationship also led to feelings of stress, frustration and loneliness, consequently seeing their partner as less than an equal. Dorothy described how she felt:

“I get stressed and irritated because, well, there's cooking to be done, laundry to take care of, bills to pay, the garden needs... everything, everything, everything, really. And you have to constantly interrupt what I'm doing to handle the next task. When he was well, he could cook for a day while I did something else, and I cooked while he did something. So… it becomes quite... yes, I find that challenging.” (Dorothy, 65)

Commonly, tending to their partner's personal hygiene and managing their incontinence were linked to descriptions of experiencing a new role in the relationship, and such tasks also made it harder to consider sexual intimacy with their partner. Participants related this to their partner becoming further dependent on them for personal care, which made it harder to feel physically attracted to them as they were less of an equal or a companion. Others expressed that personal hygiene impacted on how they perceived themselves and their role in the relationship as well. Arthur explained:

“You become like a nurse, or what I should call it. I have to help her with her hygiene, regardless of if it is her being on the toilet, I have to make sure that she washes her hands, I check that she brushes her teeth thoroughly, it is important.” (Arthur, 74)

Some participants described that caring for their partner became their new everyday life. The process of becoming a carer was described as burdensome as they were not only losing a sense of who they were, but they were also losing their partner to dementia, resulting in grief. While some showed acceptance of their new role, others expressed being uneasy with their situation as they realized that their partner's condition would deteriorate. Some related these feelings of uncertainty to suspecting that they would, sometime in the future, reach the limit of their capabilities. Some participants indicated that they had indeed reached that limit but on reaching it, the conditions became part of their everyday “normal” life, and a “new” limit was set for another future.

#### Feeling confined and forsaking own needs

A sense of confinement developed in some participants due to being committed to their partner's well-being and safety. The participants described being trapped in their carer role and situation, as their partners could not be left unattended. Some felt that they lacked the opportunity to meet others or maintain relationships or interests outside of caring. Clifford said of his situation:

“This life that I live, it is a confined life, most of the time. I cannot go out into the garden and leave her. I would not be sure of what she would do. If she would fall out of her bed since she cannot sit up for more than one hour or so. If she would fall out of bed or start to wander, I couldn’t. I always need to see her, so I stay inside.” (Clifford, 89)

Some participants described how they felt boundaries or limitations in their situation linking this to a lack of, or not being offered, support. Others mentioned how they were offered opportunities to attend different activities but that they were unable to, either due to a lack of flexibility in time or place, or not having the energy to attend. It was also described that the needs of their partner took precedence over their own, resulting in them forsaking their personal interests and needs. Others reported that they chose to forsake their own situation out of commitment or feelings of guilt. Dorothy described her situation:

“No, and the guilt that comes with it as well. Regardless, that's what I've told my children, yes, I can go and feel guilty here because I'm home and irritated. And if I were to say that now I can't handle it, now you have to live in a care facility, I would feel guilty about that too.” (Dorothy, 65)

Other participants described how they chose to focus on their own needs, as they felt that their partner could be left unattended, but that this sometimes resulted in shame. Some participants expressed their need for personal time was a cause of conflict; continuing to prioritize their own needs or interests and enduring the accompanying guilt and shame, in some instances rationalizing that they had to maintain their own activities to stay healthy, a necessity to be able to care for their partner. Others gave in to the guilt and shame and neglected their own needs. Some participants expressed that it would be better if their partner passed away rather than have their cognitive and physical abilities deteriorate further. This was perceived to be more dignified for their partner, while also allowing the carer to be relieved of their feelings of guilt. Irene described how she felt:

“Having seen what my sister-in-law went through, I worry that it will become some sort of drawn-out death. It sounds dreadful to say, but I hope it ends before reaching that stage. I wouldn’t wish that ending for anyone… To just lie there, unreachable, unable to eat, unable to do anything.” (Irene, 75)

### Longing to be seen and feel supported

Participants often expressed how their needs were conditioned by their partner's, yet they longed to be understood as an individual and spouse and not just as a carer. They also expressed how they needed to be sure that the care their partner received was of adequate quality to feel supported.

#### Valuing acknowledgement in one's individual situation

While some participants described how they were alone with caring responsibilities, some described how they still felt supported and valued that support. The sensation of being supported was often linked to people understanding their situation, such as their children, other carers or health and social care professionals. While family and friends were an invaluable support, some participants admitted being ambivalent to involving their children, as it was felt they had their own lives or that they should not be taken advantage of. Others mentioned how their children were reluctant to accept their parent's dementia or did not understand their situation. Jane described how she felt:

“I need to be in contact with people who understand this as well. Because when you say something, at least for me, it's often like, when I say something, I realize... gosh, that doesn't sound good. For me, it has been a form of survival in the process, and I need... I can't burden my daughters with some of these things, you know... let them keep the little respect they have left for their dad.” (Jane, 74)

Meeting other carers provided perspectives on their situation, resulting in a sense of being supported. This was mentioned in relation to peer support in formalized carer support groups. However, support could also be experienced in other contexts, for example, from persons with whom they had pre-existing relationships. Some described how they had friends or neighbours also caring for a partner with dementia who were able to understand the participant's situation and that their experiences mirrored the participant's own, offering a sense of affinity. Maureen described how helpful it was to be understood by peers in a support group:

“No one else understands - unless they are in this situation. They [non-carers] think, there's nothing wrong with my husband, because he can... he can talk and recognize people and all. But he has no foresight and no... can't really do anything, he does nothing at all.” (Maureen, 71)

It was reported how being acknowledged in their situation by health and social care professionals provided a sense of support. Some linked this to being valued, others that they did not burden friends or family. Irene described how a counsellor was a support for her:

“She makes me feel like she cares, and whether she truly does or if she's just very professional, I don't really care. It's the feeling that's important. That I have someone to bounce ideas off. I don't really need to worry about how she's doing; it's about me. And I don't feel like I need to burden friends so much when I can talk to someone who's a professional listener.” (Irene, 75)

In comparison, a lack of understanding or not being acknowledged resulted in frustration. Some experienced that their personal needs were not dealt with in the support group as other members had more difficult situations that required discussion. Similarly, some participants reported not feeling comfortable in a group, as it made them vulnerable when sharing their situation; or from being unreceptive to other people's hardships due to having so much to cope with themselves. Stephen described how he felt in the support groups:

“What use has the group really been? I feels as though I just sit there, giving of myself… While it was meant to be about my problems, my situation… But somehow, it feels like there is not quite enough space for me.” (Stephen, 77)

#### Needing support suitable for us

Participants seldom talked about support specifically for themselves or for their partners, rather support was received by “them”—the couple, most often home-care services, in-home respite, or day care. As such, participants' own needs for support were conditioned by their partners', and it was important for them to know that their partner was well looked after before they could feel at ease. When feeling that the care provided meant that their partner was safe, the spouse carer could hand over responsibility, giving them the opportunity to focus on their own interests or needs. Arthur explained how he felt when his wife was at day care:

“Then, the time between when she goes, and she comes home, I am completely free. I don’t have to… I don’t feel the least concerned or worried about her and what will happen to her. I know she is taken care of at the day care centre, and it works well, so I can relax completely.” (Arthur, 74)

How professional dementia care was organized, and the perceived quality of care, were important for participants. Professionals' ability to provide the participant with a sense that they and their partner were in safe hands was linked to how the support was organized. Participants described how staff continuity was important for both their own safety and their partner's, and how high staff turnover or different staff for day care and respite care affected their sense of safety. Dorothy noted:

“…We could get home care and all that, but it's my home as well. I don't want to have umpteen different people coming in here every week to help him, and... no, it feels... no, it doesn't feel secure.” (Dorothy, 65)

Others described how it was hard to make decisions on formal care for their partner and that they seldom had a need to be apart from their partner, rather it was the absence of responsibility for their partner's dementia and well-being that they longed for.

## Discussion

This study revealed that spouse carers of persons with dementia experienced that their everyday life was consumed by caring, that they became lost in the carer role, felt confined to the home and that they had to deal with a new everyday life conditioned by their partner's dementia. Further, findings revealed that they wanted to be seen and understood not only as carers but also as individuals with their own needs, and that support must meet the needs of not only the carer but also their partner.

Research on spouse carers of persons with dementia has reported that they may lose the sense of who they are as they become overwhelmed by their caring responsibilities. This suggests that they may need support to adapt to their situation and ease the transition into a caring role (Miller et al., 2025; Steenfeldt et al., 2021; Zhu et al., 2024). However, while our results indicate that spouses have to navigate an uncertain situation and may need to adapt, they may not necessarily want to accept their situation. Spouse carers must deal with their situation due to a lack of alternatives, but they may still wish for a different situation or for opportunities to have time away from caring to maintain their own activities and interests necessary for their well-being.

Further, our results showed that spouse carers can experience that they are confined in their caring situation and their carer role. This finding echoes a previous report by Lee et al. (2022). Our findings suggest that experiencing limitations in everyday life was linked to a complex interplay of shame, guilt, and perceptions that they were unable to leave their situation out of commitment to their partner, or fear that without them their partner was at risk of harm. As a result, spouse carers sometimes chose to forsake their own needs or interests, or to live with the guilt if they address their own needs as it was essential that their partner was being cared for and was safe. Similar results have previously been reported by Miller et al. (2025) and Tolhurst et al. (2019), who argue that the interdependence of needs in couples affected by dementia requires recognition. Similarly, other studies suggest that spouse carers may need support to reconcile their own personal needs with their perceived care responsibilities (Engel et al., 2022; Shiff et al., 2025; Zhu et al., 2024). These findings emphasize that spouse carers should be considered active care recipients as well, as reflected in our results where participants often described that the “couple” received support and that their needs were conditioned by their partners' needs, functional abilities, or dementia. Other studies have reported on the significance of how care is perceived by carers of persons with dementia, as it affects receipt of both carer support and formal care for the partner, and that both carer and partner prefer to be involved in the decision-making and care processes as dementia progresses (Balvert et al., 2024; Bannon et al., 2022; Pope et al., 2025). Our findings, that spouse carers experience a loss of companionship and intimacy with their partner while also having shared needs with their partner for care and support, argue for more couple-centred support. Approaches that aim to strengthen the spouse carer and partner's couplehood should be beneficial for both the caring spouse and the partner with dementia (Albert et al., 2022; Landolt et al., 2023; Miller et al., 2025; Stefánsdóttir et al., 2022).

The shared needs and the precedence of their partner's needs may also be linked to a loss of self in the carer, as spouse carers tend to solve the tension between their own and their partner's needs by prioritizing their partner's needs (Miller et al., 2025; Morgan et al., 2021; Zhu et al., 2024). Similarly, our results show that spouse carers experience a new role, which could be understood as a transitioning of their identity from spouse to carer (Montgomery & Kosloski, 2009). However, caregiver identity theory has been criticized as deterministic for implying that the transition to the carer role is inevitable. Studies have found that some carers resist identifying as a carer (Knowles et al., 2016; Morgan et al., 2021), which may reduce their support-seeking behaviour. Providing care may seem like part of the role and duty of a spouse who is thus not eligible for formal support (Beatie et al., 2021; Knowles et al., 2016; Miller et al., 2025).

Our results also showed that caring for a partner with dementia not only affected their own identity and spousal role but also their perceptions of their partner's role in the relationship. This supports findings from studies that perceiving oneself as carer may cause tension between self-identity and individual needs, and the preservation of their partner's personhood (Duggleby et al., 2017; Zhu et al., 2024). The change in the relationship is also reflected in our finding that spouses experienced a loss of companionship and intimacy with their partner with dementia. The importance of the spousal relationship is a known factor affecting spouse carers (Miller et al., 2025; Swall et al., 2020). As discussed above, our findings indicate that spouse carers may long for a different situation, and the rejection of a carer identity might underly our finding that spouses need to be understood and valued as individuals and not only as carers.

### Implications for policy and practice

Our study highlights the importance of health and social care professionals' ability to make spouse carers feel visible and secure, and practice should focus on forming an alliance with both the spouse carer personally and the couple collectively to increase support uptake and thereby meet the couples joint support and care needs. A support group is one of the most commonly offered types of support to spouse carers (Johansson et al., 2021). Our findings suggest that support groups should focus on the priorities set by the participating spouse carers, as they will have differing situations or want to raise topics that could be regarded as sensitive, for example, couple intimacy. It may also be important that the groups are based on similarities between participants, as our results show that spouse carers can reject or withdraw from groups with which they do not identify.

### Methodological considerations

One limitation of this study is that data were collected during the COVID-19 pandemic, and participants' situation at the time was influenced by the recommendations of social distancing that were in place in Sweden (e.g. as described by Rokstad et al. (2021)). These recommendations also meant that the interviews had to be conducted by phone. While phone interviews make it harder to discern non-verbal communication and reactions, such interviews have been found to make participants comfortable when discussing sensitive topics (Mealer & Jones, 2014). We noticed that most participants were eager to share their experiences of caring for and living with a partner with dementia and, even though some participants expressed that it was hard to talk about their situation or would become emotional, they wanted to complete the interview.

Thematic analysis was chosen, as it allowed a combined approach to the material using both semantic and latent understandings and interpretations of data; further, Braun and Clarke (2022) approach also provides structure to the analysis process. The first author, a qualified social worker, conducted the coding of the transcripts, and this process was reviewed and discussed among the researchers involved in the analysis. Trustworthiness was also strengthened as theme development was performed in collaboration with two of the co-authors, both registered nurses and senior academic scholars with expertise in dementia care, care of older adults and carer support. The process of theme development and interpretation was also audited by the remaining members of the research team, and any inconsistencies and disagreements in interpretations were discussed until consensus was reached.

The study included many participants who provided rich interviews, offering robust material for analysis and a sample that was diverse on several characteristics such as carer age, duration of caring, and dementia diagnosis. However, most participants were women, which reflects that it is more common for men than women to receive care from a partner (Dahlberg et al., 2018) but may also reflect gendered patterns of engagement in research reported in previous studies (Borg et al., 2024). Still, more knowledge on men's caregiving and gendered norms in informal care is needed. Furthermore, the eligibility criterion concerning proficiency in Swedish will have excluded some spouse carers from migrant or ethnic minority backgrounds, while the sample included only one spouse carer in a same-sex relationship. Future studies could explore the experiences of spouse carers from minority groups, as findings reported in this study may not reflect the experiences of persons from such groups. There are also additional topics related to caregiving that should be further explored, for example, the consequences for carers' health from disrupted sleep patterns and health behaviours such as exercise.

## Conclusions

In conclusion, our study emphasizes the importance of aligning the support provided by health and social care professionals with the specific needs of spouse carers of persons with dementia. Spouse carers can often feel trapped in their caring role and put their partner's needs before their own, experiencing guilt or shame if they do not do so. They also experience a loss of intimacy with their partner, becoming more of a carer than a spouse. For spouse carers to feel adequately supported, health and social care professionals should focus on preserving the spouse's sense of self while simultaneously embracing a couple-centred approach, so that spouse carers feel acknowledged as individuals and as part of a couple, as well as more secure in the challenging carer role they undertake.

## Supplementary Material

Supplementary Material SM1 Interview guide.docx

## Data Availability

The participants of this study did not give written consent for their data to be shared publicly, so due to the sensitive nature of the research supporting data is not available.
