# Supplementary material for: Exploring the experiences and needs in everyday life of spouse carers of persons with dementia
Source: Int J Qual Stud Health Well-being. 2026 May 27;21(1):2680726. doi: 10.1080/17482631.2026.2680726 (PMC13220580; doi:10.1080/17482631.2026.2680726)
Supplement: Supplementary Material — SM1 Interview guide.docx [file ZQHW_A_2680726_SM2650.docx]

| **Interview area** | **Topics and suggested probing questions** |
| --- | --- |
| **Relationship** | ***How do you experience your relationship after the onset of your partner’s dementia disease?***  ***What are the major differences in the relationship compared with before the onset of your partner’s dementia?***  ***How do you express your feelings toward each other, both negative and positive feelings?***  ***Are you able to talk about your feelings? If so, how?***   - When you feel happy? - When you feel sad?   ***How do you express tenderness towards each other?***   - How do you express tenderness? - How does your partner express tenderness?   ***Is your relationship with your partner intimate?***   - In what way? - Do you have an active sex life with your partner? |
| **Life situation** | ***Could you describe your everyday life?***   - Are there things you like to do that you are unable to do these days? - Is that because of having to care for your partner??   ***How would you describe your health?***   - What could improve your health?   ***Would you say that you are lonely?***   - What do you do when you feel lonely?   ***What are the most difficult or stressful situations in your everyday interactions with your partner?***  ***What are the most difficult or stressful situations when providing care to your partner?***  ***How do you feel in situations when you have disagreements or do not understand each other?***   - How do you act/react? What helps to manage the situation? - How does your partner act/react in those situations? How does your partner seem to experience/feel in those situations? |
| **Future** | ***How do you feel about your own life journey?***  ***Do you think about what your life has come to, and wonder about what it will be like for you in the future?*** |
| **Support** | ***What type of professional support do you have today, for yourself or as a couple?***   - What do you think about the support you receive? - Could you describe the pros and cons of the support you receive?   ***What type of support would you like to receive?***   - For your partner? - For yourself? - How would that improve your situation?     ***What type of emotional or psychosocial support would you like to receive for yourself?***   - How do you think such support could benefit you?   ***What is your opinion of the following forms of support?***   - Web-based support or chat forum with others in your situation? - Web-based support or chat forum with different health care professionals? - Web-based information with educational films or literature about support? - Physical support groups with others in your situation? - Individual face-to-face counseling sessions with a health care professional? - Meeting spaces to spend time with your partner and other couples affected by dementia?   ***What do you think are the most important things about how support is delivered?***  ***Are there other forms of support you would like to receive?*** |
| **Ending** | ***Is there anything you would like to add that you think is important for understanding your situation that has not been mentioned?*** |
